# Movement and Frontal Theta Activity During Step-Based Exergaming in Healthy Younger and Older Adults: Laboratory-Based Cross-Sectional Study

**DOI:** 10.2196/100270

**Published:** 2026-10-06

**Authors:** Nina Skjæret-Maroni, Helen Müller, Phillip Anders, Karoline Blix Grønvik, Jochen Baumeister, Beatrix Vereijken, Ellen Marie Bardal

**Affiliations:** 1 Department of Neuromedicine and Movement Science Faculty of Medicine and Health Sciences Norwegian University of Science and Technology Trondheim, Trøndelag Norway; 2 Exercise Science and Neuroscience Unit Faculty of Science Paderborn University Paderborn, North Rhine-Westphalia Germany; 3 IT Department Melhus Municipality Melhus, Trøndelag Norway; 4 The Rehabilitation clinic St Olav's University Hospital Trondheim, Trøndelag Norway

**Keywords:** Exergames, Frontal theta, Rehabilitation, Movement, Age groups

## Abstract

**Background:**

Exergames are increasingly used in rehabilitation to simultaneously target physical and cognitive functions. Game scores are commonly used as indicators of performance, yet it remains unclear to what extent they reflect underlying movement and cognitive-control demands. Understanding this relationship is essential for interpreting what exergame performance scores reflect during gameplay.

**Objective:**

We aimed to investigate how movements and frontal theta activity, used as a proxy for cognitive-control demands, relate to game performance across game levels and age groups in a step-based exergame.

**Methods:**

This cross-sectional laboratory study included 32 participants (16 older adults: mean age 76.5, SD 4.5, range 71-86 years; 16 younger adults: mean age 25, SD 2.1, range 22-28 years) recruited using convenience sampling from a university and recreational exercise groups in Trondheim, Norway. Participants completed a step-based exergame (“The Mole”) at 3 difficulty levels (easy, medium, and hard). Movements were measured using accelerometers (vector magnitude and step count), and frontal theta activity was derived from mobile EEG (electroencephalogram) as mean power spectral density in the 4-7 Hz range as a proxy for cognitive-control demands. Game scores were recorded as performance indicators. Repeated-measures general linear models were used to test effects of difficulty and age group, and Pearson correlation analyses examined associations between variables.

**Results:**

Younger adults achieved higher game scores than older adults across all difficulty levels, and performance declined at higher task difficulty (all *P*<.001). The amount of movement (vector magnitude) showed strong and generally consistent positive correlations with game score across all levels and both age groups (*r*=0.67-0.80). All correlations except the hard level in older adults were statistically significant after correction for multiple comparisons. Step count showed weaker and less consistent associations across groups. No statistically significant differences in frontal theta activity were observed between age groups or across task difficulty levels (all *P*>.15). Associations between frontal theta and game score were near 0 in younger adults (*r*=−0.096 to 0.001), whereas older adults showed small-to-moderate correlations at easy and medium difficulty levels (*r*=0.345-0.416) and a stronger correlation at the greatest difficulty level (*r*=0.76). However, none of the correlations between frontal theta and game score were statistically significant after correction for multiple comparisons (all *P*>.003).

**Conclusions:**

In this controlled exergaming setting, game performance was strongly associated with the amount of movement performed, whereas associations with frontal theta activity, used as a proxy for cognitive-control demands, were limited and context-dependent. These findings suggest that game scores primarily reflect movement output rather than cognitive-control processes under the present game conditions. The results indicate that game scores alone may provide an incomplete representation of engagement and highlight the importance of considering both physical and cognitive aspects when designing and evaluating exergames.

## Introduction

At present, an estimated 2.4 billion people are living with various health conditions that may benefit from rehabilitation [1]. Rehabilitation of such health conditions is a key element for achieving the World Health Organization’s third sustainable development goal: “Ensure healthy lives and promote well-being for all at all ages.” However, the increasing life expectancy in combination with a shortage of qualified health care workers in the years to come poses significant challenges to achieving this goal. According to the current National Health and Hospital plan for Norway, digitalization, coupled with technological and organizational innovations, are proposed solutions to bridge the gap between the increasing health care demands and the shortage of health care staff [2]. Digitalization in health care has increased and reshaped health care services in recent decades [3], as it can improve access and reduce barriers to treatment [4]. Digital practice in rehabilitation is becoming more viable and has been widely implemented, especially after the COVID-19 pandemic [5-8]. Digital practice in rehabilitation enables interaction between rehabilitees and health care personnel residing at different geographical locations, connecting through digital technologies either in real time or asynchronously, and can also offer feedback and support to the rehabilitees during the rehabilitation process [9].

Exergames, or exercise-based video games, are an example of a digital tool that is increasingly explored and used in treatment and rehabilitation in children [10], and younger [11] and older adults [12,13] with a variety of physical disabilities. Exergames require movement from the player in an interactive and cognitively demanding digital, augmented, or virtual game-like environment [14]. These games typically require players to perform movements such as stepping, jumping, or reaching [14-16], often in response to visual or auditory cues [17,18]. By combining movements with game-like challenges, exergames offer an engaging alternative to traditional therapy, enhancing motivation and adherence [19] while supporting higher movement dosage [20-22]. Additional benefits include reduced boredom [23], opportunities for dual-task training [24], and improvements in cognitive function, particularly in older adults [25]. In rehabilitation, the overarching goal is to restore lost functions, activities, and roles by promoting functional capacity and participation. Exergame-based interventions can be designed to target core rehabilitation objectives such as weight transfer, balance control, joint loading, and muscular coordination [26-28]. However, the value of exergames extends beyond their physical demands. Many incorporate cognitive elements such as decision-making, information processing, and rapid responses, thereby simultaneously training motor and cognitive functions [14,29,30]. This integrated physical-cognitive approach is especially relevant for older adults, who may benefit from combined training strategies to maintain or improve functional independence. Consequently, exergame-based training holds substantial potential not only as a rehabilitation tool across a range of pathologies, but also for health promotion and disease prevention across different populations and age groups [24,31].

Despite their increasing use, there is limited understanding of how exergames influence physical and cognitive activity across different age groups. Previous research has primarily focused on energy expenditure and general activity levels [32-35], while only a limited number of studies have examined movement characteristics during gameplay in parallel with neurophysiological indicators such as brain activity. A small but growing body of work has begun to address this relationship, demonstrating that exergame characteristics can influence both physical behavior and cortical activity during gameplay [35,36]. However, a recent systematic review indicates that studies combining objective movement measures and EEG (electroencephalogram) during exergaming remain limited [37]. This gap is particularly evident in relation to specific game design features, such as difficulty level and feedback mechanisms, which are central to how exergames are used therapeutically. In both clinical practice and research, game scores are commonly used as outcome-based indicators of performance and as feedback to the player and are often assumed to reflect both physical and cognitive activity [35,38]. Importantly, game scores should be distinguished from game difficulty level, which represents a design feature of the task that is manipulated to alter physical and cognitive demands. While difficulty level defines the constraints under which the task is performed, the score reflects how well the player performs within those constraints. However, this assumption has not been tested against objective physiological measures. Exergames are often described as combining physical and cognitive demands; however, the extent to which cognitive engagement is involved varies considerably depending on game design features such as task complexity, stimulus characteristics, and interaction requirements. Recent conceptual frameworks emphasize that exergames are heterogeneous interventions that can range from predominantly motor-focused to more integrated motor-cognitive tasks [39,40]. Studies further show that cognitive and physical outcomes may vary depending on specific game design features and training approaches [37,41]. Accordingly, cognitive demands should not be assumed but rather empirically examined in relation to specific game characteristics and performance measures [42]. Motivation during gameplay is often driven by tracking progress and receiving rewards for performance, which reinforces engagement and supports skill development [43]. Accordingly, game design features that provide clear and meaningful feedback on progression and effort are thought to strengthen the sense of mastery and enhance engagement [36,44]. If feedback mechanisms, such as game scores, fail to accurately reflect player effort or task complexity, engagement may decline rapidly [35,45]. From a rehabilitation perspective, this underscores the importance of understanding what game scores capture in terms of underlying physical and cognitive activity. To achieve specific rehabilitation goals, knowledge about how the game dynamics support moment-to-moment engagement of the player with the game is critical [44]. Previous research has shown that game characteristics and game settings, such as the different difficulty levels or game speed [36,46] and additional cognitive elements in the game [45], influence how people move when playing games. However, much less is known about how these design features modulate cognitive engagement during exergaming, or how cognitive demands interact with movement execution.

To address this gap, objective markers of cognitive engagement are needed. In this study, we therefore use frontal theta EEG activity as a physiological proxy for cognitive-control demands. Frontal midline theta oscillations (4-7 Hz) are strongly associated with attentional control, working memory demands, conflict monitoring, and other core executive processes [47]. Importantly, increases in frontal theta have also been observed during cognitively demanding motor tasks and exergaming, suggesting that it is associated with the integration of cognitive control and movement execution [35,48]. As such, frontal theta provides a complementary, objective index of the cognitive demands imposed by specific game design features (eg, speed, distractors, or inhibitory elements), beyond what can be inferred from performance alone. The next essential step is therefore to clarify the relationship between game performance and the underlying physical and cognitive activity elicited during exergaming. If game scores are to be used as indicators of performance in clinical and research settings, it is important to clarify which underlying dimensions they reflect. In the context of exergaming, player performance and engagement may relate to multiple aspects, including the amount of movement performed, qualitative aspects of movement (eg, coordination or balance), and cognitive engagement. In the present study, we specifically focus on the amount of movement, assessed using accelerometry (vector magnitude [VM] and step count), and cognitive-control demands, indexed by frontal theta EEG activity. Movement quality was not assessed and is therefore beyond the scope of this study. Therefore, the aim of this laboratory-based experimental study is to investigate how game scores relate to the amount of movement performed and frontal theta activity across difficulty levels and age groups. This knowledge is crucial for informing the design and clinical application of exergames in rehabilitation settings, ensuring that they support meaningful and measurable improvements in health outcomes.

## Methods

### Study Design

This study used an experimental cross-sectional design to investigate frontal brain activity and physical activity during exergaming in younger and older adults. Data were collected at the NeXt Move Core Facility at the NTNU (Norwegian University of Science and Technology), Trondheim, Norway.

### Participants

#### Inclusion and Exclusion Criteria

Participants were eligible if they were either between 20 and 30 years of age or above 70 years of age and community-dwelling. Participants were excluded if they had a history of neurodegenerative and/or neurologic diseases, acute physical or mental problems that prevented them from playing an exergame safely, or surgery or injury to the back or lower extremities that affected their ability to move without a walking aid.

#### Participant Characteristics

Thirty-two participants were included in this study, comprising 16 younger adults and 16 older adults. The younger adults had a mean age of 25.0 (SD 2.1, range 22-28) years, while the older adults had a mean age of 76.5 (SD 4.5, range 71-86) years. Both groups consisted of 8 women and 8 men. Participant characteristics are presented in Table 1.

**Table 1:** Participant characteristics for younger and older adults. Group differences were assessed using Mann-Whitney U tests, with effect sizes reported as Cohen *d*.

	Older adults (n=16)			Younger adults (n=16)			Group differences	
	Mean (SD)	Range	Mean (SD)		Range	*P* value		Cohen *d*
Age (years)	76.5 (4.5)	71-86	25 (2.1)		22-28	<.001^a^		—^b^
Height (cm)	171.9 (9.3)	159-194	175.9 (9.7)		158-191	.22		0.42
Weight (kg)	75.8 (15.6)	60.8-128	71.1 (12.2)		47.4-90	.69		0.34
BMI (kg/m²)	25.5 (2.9)	22.7-34	22.9 (2.9)		18.5-27	.02		0.91
Self-reported physical activity (min/wk)	99.4 (12.1)	75-105	99.4 (12.1)		75-105	>.99		0.00

^a^Significant group differences.

^b^Not applicable.

#### Sampling Procedures

A convenience sampling approach was used. Younger adults were recruited from the university community, and older adults from recreational exercise groups in the municipality of Trondheim, Norway. Interested individuals received information about this study and were screened against the eligibility criteria before participation.

#### Sample Size, Power, and Precision

As this study was designed as an exploratory laboratory investigation, a formal a priori power analysis was not conducted. The intended sample size (16 younger and 16 older adults) was determined pragmatically based on feasibility considerations and consistency with previous mobile EEG studies using similar exergaming paradigms. Consequently, this study was not designed to provide definitive evidence regarding the absence of group differences and should be viewed as hypothesis-generating.

### Ethical Considerations

This study was approved by the Regional Ethical Committee for Medical and Health Research Ethics in Norway (2018/45) and was conducted in accordance with the Declaration of Helsinki. All participants provided informed, written consent before entering this study. Participant data were deidentified before analysis to ensure confidentiality and privacy. Participants did not receive financial compensation for participation. No images in this paper contain identifiable individuals.

### Measures and Covariates

The primary outcome measures were game performance score, movement characteristics, and frontal theta activity. Age group was included as a between-participant factor in the analysis.

#### Exergame

A step-based exergame, The Mole, which is recommended for balance training in physical rehabilitation of older adults, was chosen for this study. The game is played on a television-based exergame system (SilverFit BV 3D, Netherlands). The system uses a time-of-flight camera to control the game, which records the body movements of the player in 3 dimensions within a 5 × 5 meter game area [49]. The game was played at 3 levels of difficulty: easy, medium, and hard. These difficulty levels represent predefined task constraints designed to systematically manipulate the physical and cognitive demands of the game, independent of the performance score. At the easy level, the goal is to step on stationary moles as they appear on the screen. At the medium level, players should step on both stationary moles and moving mice, while at the hard level, players also need to avoid stepping on additional stationary ladybugs (Figure 1). All creatures appear randomly on the screen, prompting the player to move in all directions. The game score is based on the number of animals hit and/or avoided. The moles and mice yield 1 point each, while hitting a ladybug costs 2 points.

**Figure 1:**
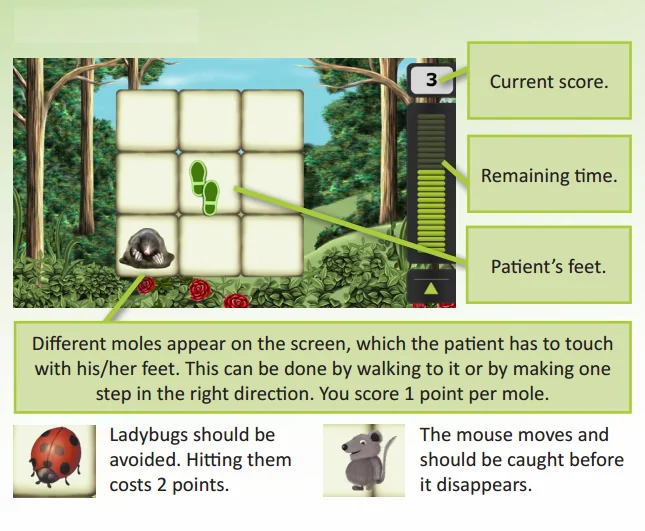
Screenshot of The Mole game with explanation of what the players see on the screen and game scores.

#### Performance Score

Game performance was assessed using the total game score recorded at the end of each trial. Total game score was registered after each trial and consisted of the number of moles hit (for all 3 difficulty levels), the number of mice hit (medium and hard levels), minus the number of ladybugs hit (hard level). Total game score was used as the primary performance outcome measure across all difficulty levels.

#### Movement

Movement was assessed using a triaxial accelerometer (AX3, Axivity, United Kingdom) positioned on the lower back (L3). VM (vector magnitude), representing the overall amount of movement performed during gameplay, and number of steps, representing stepping activity during gameplay, were derived from the accelerometer recordings.

#### Frontal Theta Activity

Frontal theta activity was assessed using a mobile 64-channel EEG system (MOVE and actiCHamp & actiCAP, Brain Products, Germany). Frontal theta activity (4-7 Hz) was selected a priori as a neurophysiological proxy for cognitive-control demands during gameplay. Theta power was derived from a frontal independent-component cluster and expressed as mean spectral power density (µV²/Hz). A total of 21 participants contributed to the final frontal cluster (11 older adults and 10 younger adults).

#### Data Collection Procedures

All participants were invited to an information session in the laboratory to familiarize themselves with the equipment and laboratory environment before data collection took place. Before testing, participants were asked to fill out a background questionnaire covering age, previous gaming experience, and self-reported physical activity based on items from the Helseundersøkelsen i Trøndelag questionnaire, which combines information regarding exercise frequency, intensity, and duration to estimate weekly physical activity. Additionally, weight and height were measured. Subsequently, participants were fitted with the active 64-channel EEG system (MOVE, actiCHamp &actiCAP) to record brain activity, and a triaxial accelerometer (AX3) was placed on the lower back (L3) to measure movements during the gameplay.

All participants completed 2 gameplay trials at all 3 difficulty levels. Each trial lasted 5 minutes, giving a total of 30 minutes of gameplay for each participant. The game was demonstrated and explained to the participants before the trials. There was a 1-minute break between each trial and a 2-minute break between the levels. The first trial at each difficulty level was considered a test trial for all participants to familiarize themselves with the game and was excluded from further analysis. The order of the 3 levels was counterbalanced across all participants and sessions. One researcher always stood near the participants to ensure safety while playing the exergame (see Figure 2 for gameplay setup).

**Figure 2:**
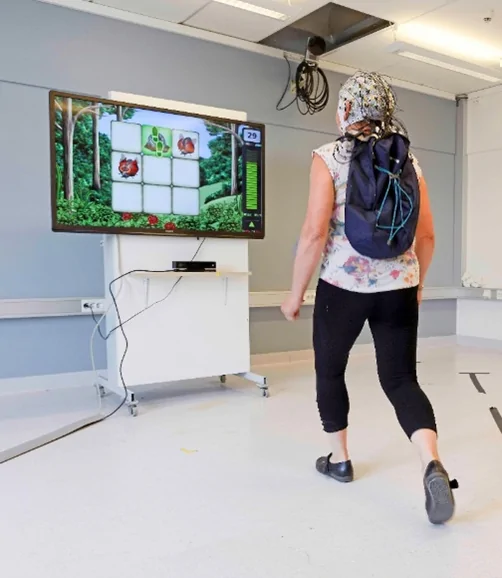
Experimental setup showing the gameplay setup.

### Data Processing

#### Movement

Acceleration was sampled at 100 Hz (range ±8 g). Mean VM of the lower back acceleration and number of steps were calculated as measures of the amount of movement participants performed during the games. VM was calculated using the Euclidean norm of the high-pass filtered (fourth-order Butterworth 0.2 Hz high-pass filter) triaxial acceleration signal method [50]. Data were averaged over consecutive nonoverlapping 10-second time windows. Steps were identified by detecting local minima in the vertical acceleration signal. Data were filtered using a fourth-order Butterworth band-pass filter with a passband of 0.2-15 Hz. One young participant was excluded from all accelerometry analysis due to technical problems during data collection and insufficient signal-to-noise ratio. In addition, total step count could not be calculated for 2 participants because data from 1 foot-mounted accelerometer were unavailable. Consequently, VM analyses were based on 31 participants, whereas step-count analyses were based on 29 participants.

#### Frontal Theta Activity

Frontal theta activity was recorded using 64 active EEG electrodes placed in a tight-fitting cap (actiCAP) connected to transmitters stored in a small backpack that sent the signals wirelessly to an amplifier (MOVE and actiCHamp). The sampling rate was set to 1000 Hz. Using the 10-10 extension of the international 10-20 system [51], the EEG electrodes were placed with the ground electrode on the midforehead at the anterior frontal midline electrode [52] and referenced online to frontocentral midline electrode position. The impedance was kept below 5 kΩ to ensure sufficient signal-to-noise ratio. Triggers were set manually when starting and stopping each condition.

EEG data were processed using the EEGLAB toolbox v2020_0 [53] for MATLAB (version R2019b, Mathworks Inc). An EEG processing pipeline was used following previous studies [35,48,54]. Sinusoidal noise was removed using the CleanLine plug-in [55], before finite impulse response filtering the data at 3 and 30 Hz. Finally, the data were rereferenced to a common average and downsampled to 256 Hz. By applying the eBridge plug-in [56], channels that were linked via electrical bridges were detected and removed. Remaining noisy channels were deleted using the EEGLAB pop_rejchan function with a threshold of 5 SD. On average, 49.3 (SD 2.67) of 64 channels were kept per participant. Further cleaning of artifacts was done by using the clean_rawdata EEGLAB plug-in [57]. Data with nonstereotypical artifacts or significant noise were removed, and large-amplitude artifacts were interpolated using automated subspace reconstruction (ASR) with SD set to 7 as the cutoff value, as used in previous studies [58,59].

The cleaned data were separated into independent components (ICs) using the AMICA (Adaptive Mixture Independent Component Analysis) algorithm (runamica15) implemented in EEGLAB [60]. AMICA was run with a single mixture model (num_models=1). No user-defined modifications of the default AMICA parameters were applied. Subsequently, the dipoles for each IC were calculated using the DIPFIT toolbox [61]. The IClabel plug-in [62] assigned each IC to brain signals or nonbrain signals (muscle activity, eye activity, EKG [electrocardiogram], line noise, and channel noise). All ICs that were classified as brain-related with a probability greater than 90% and with dipoles located within the head model and a residual variance below 15% were retained for further analysis. The decomposition of the remaining channels resulted, on average, in 18.92 (SD 3.66) functional brain components per participant, 2.63 (SD 2.51) labeled as muscle activity, 2.7 (SD 1.5) as eye movements, 0.83 (SD 1.09) as EKG, none as line noise, 2.97 (SD 2.24) as channel noise and 21.23 (SD 4.08) as others. For the source-based approach, the brain ICs were clustered according to their dipole location and orientation, power spectra, and scalp maps into 5 clusters (1 frontal, 2 central, and 2 parietal clusters). Outliers were defined as dipoles with an SD greater than 3 from the mean dipole of the final cluster. Given the a priori hypothesis regarding frontal theta activity as an index of cognitive engagement, only the frontal cluster was included in subsequent analyses. If participants contributed more than one IC to the frontal cluster, the IC with the most frontal dipole location was used for further processing. The frontal cluster is presented in Figure 3.

**Figure 3:**
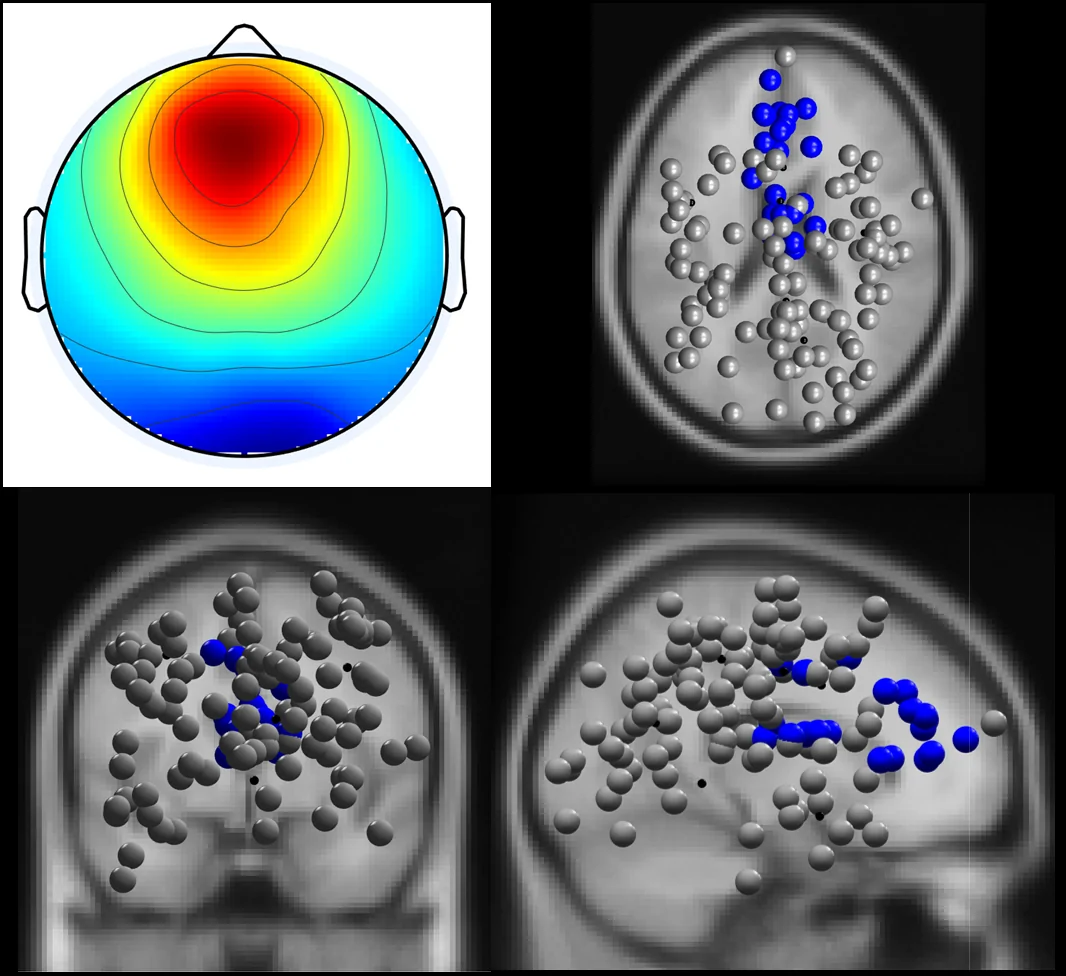
Dipoles from coronal, sagittal, and top views and the scalp map of the frontal cluster. A total of 21 participants (11 older and 10 younger adults) were included in the cluster, and the mean residual variance (RV) was 7.27%.

Frontal theta activity was derived from the frontal IC cluster, including 21 participants (n=11 older adults and n=10 younger adults). All subsequent EEG analyses were based on this subset. Spectral power data (specdata) were exported at the single-frequency-bin level for each IC and condition. Theta power was defined as the mean spectral power across the 4-7 Hz frequency range. For each participant and condition (easy, medium, or hard), spectral power values within this frequency range were averaged to obtain a single frontal theta measure for subsequent statistical analyses.

### Statistical Analysis

Visual inspection of histograms and Q-Q plots, along with the Shapiro-Wilk test, indicated that all variables (game scores, step counts, movement, and EEG measures) were within a normal distribution, supporting the use of parametric analyses.

Means and SDs within each group were computed for demographic and physical activity variables. Group differences were examined using the Mann-Whitney *U* test. To examine the effects of difficulty level and age group on movement measures and frontal theta activity, repeated measures general linear models were used with difficulty level as a within-participant factor and age group as a between-participant factor. This approach accounts for within-participant dependency in repeated measurements in a balanced design. Sphericity assumptions were checked, and Greenhouse-Geisser corrections were applied where necessary. The significance level was set at *P*<.05.

Pearson correlation analyses were performed to explore associations between movement, step count, frontal theta EEG activity, and game performance scores. Correlations were computed for younger and older adults separately. To control for multiple comparisons, a Bonferroni correction was applied. In the present analysis, 18 correlations were performed, giving a corrected significance level of *P*>.003. Only correlations exceeding this corrected threshold were considered statistically significant. All statistical analyses were performed with IBM SPSS Statistics (version 30).

## Results

### Participants

A total of 32 participants (16 younger and 16 older adults) completed this study. Descriptive statistics for participant characteristics are presented in Table 1. Both groups had equal sex distribution (8 women/8 men). Seven of the older adults had some previous experience with exergaming consoles, while all the younger adults had tried exergames before, mainly Wii Sports and EyeToy. None of the participants used exergames on a regular basis. Both the younger and the older adults reported similar levels of physical activity, being physically active 4-5 times per week for approximately 90 minutes per week with medium to high intensity.

### Performance Scores

The performance scores showed that younger adults significantly outperformed the older adults at all levels (Figure 4A). At the easy level, younger adults had a mean score of 175 (SD 19.7, range 134-201) points, while older adults had a mean score of 142 (SD 13.6, range 112-164; *P*<.001). Similar results were observed at the medium (175, SD 20.5, range 146-209, vs 138, SD 14.1, range 115-166, respectively) and hard (158, SD 20.7, range 119-190, vs 108, SD 18.7, range 62-134, respectively) levels (all *P* values <.001). There were significant effects of age group (*F*_1,30_=46.57, *P*<.001, ηp²=0.61) and difficulty level (*F*_2,60_=82.97, *P*<.001, ηp²=0.73), as well as a significant interaction between them (*F*_2,60_=7.89, *P*<.001, ηp²=0.21).

**Figure 4:**
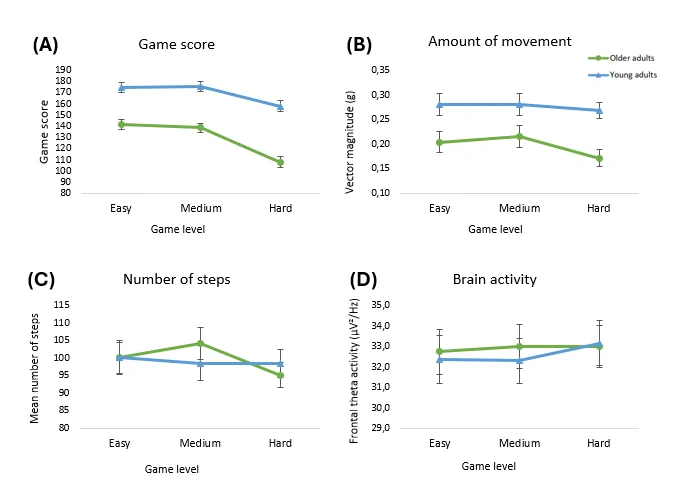
Mean game score (±SE) (A), mean amount of movement estimated as vector magnitude (VM; ±SE, g) (B), mean number of steps (±SE) (C), and mean frontal theta activity (±SE, µV²/Hz) (D) for older and younger adults at easy, medium, and hard game level. Panel (A) is based on 32 participants. Panel (B; VM) is based on 31 participants (1 excluded due to missing accelerometry data). Panel (C; step count) is based on 29 participants (2 additionally excluded owing to missing data from 1 foot-mounted accelerometer). Panel (D) is based on 21 participants (11 older and 10 younger adults contributing to the frontal EEG [electroencephalogram] cluster).

### Movement and Steps

Difficulty level affected the amount of movement (measured by VM) during gameplay for both younger and older adults (Figure 4B). There was a significant main effect of difficulty level on the amount of movement (*F*_2,58_=6.690, *P*=.002, η²=0.187), indicating that movement amount decreased as game difficulty increased. In general, younger adults displayed more total body movement than older adults (*F*_1,29_=7.931, *P*=.009, η²=0.215), particularly at the hard level. However, there was no significant interaction between difficulty level and age group (*F*_2,58_=1.326, *P*=.274, η²=0.044).

For the total number of steps made during a gaming trial (n=29), there was no statistically significant main effect of game level on step count (*F*_2,54_=2.655, *P*=.079, η²=0.090), although a trend toward fewer steps at higher difficulty levels was observed. There was also no significant main effect of age group (*F*_1,27_=0.015, *P*=.902, η²=0.001), indicating similar overall step counts between younger and older adults. Furthermore, there was no significant interaction between age group and difficulty level (*F*_2,54_=2.471, *P*=.094, η²=0.084), indicating that both younger and older adults showed similar patterns of step count across levels (Figure 4C).

### Frontal Theta

Mean frontal theta values (n=21) were similar between younger adults (easy: 32.35, medium: 32.29, and hard: 33.15) and older adults (easy: 32.72, medium: 32.98, and hard: 32.99; Figure 4D). There was neither a significant main effect of game level on frontal theta activity across easy, medium, and hard conditions (*F*_2,38_=1.96, *P*=.155, η²=0.094), nor a main effect of age group (*F*_1,19_=0.04, *P*=.844, η²=0.002). Likewise, there was no interaction between difficulty level and age group (*F*_2,38_=1.11, *P*=.340, η²=0.055).

### Correlation Analyses

The amount of movement (VM) during the game shows strong, positive correlations with game scores for both age groups and across all difficulty levels (range 0.67-0.80; Figure 5). Five of the 6 VM correlations remained statistically significant following Bonferroni correction (all *P*<.003), whereas the correlation at the hard level for older adults (*r*=0.67; *P*=.04) did not. For younger adults, the number of steps taken during the game shows moderate, positive correlations with game score across game levels (from easy to hard, *r*=0.59 to r=0.40; *P*=.03-.20). For older adults, number of steps shows moderate correlations with game score for the easy level (*r*=0.52; *P*=.04), with weaker correlations observed at the medium (*r*=0.35; *P*=.18) and hard (*r*=−0.13; *P*=.18) levels. None of the correlations between step count and game score remained statistically significant following Bonferroni correction (all *P*>.003). Frontal theta EEG showed near-0 associations in younger adults across all levels (from easy to hard, range 0.001 to −0.096; *P*=.86-.99). In older adults, correlations ranged from 0.35 to 0.76 (*P*=.02-.24). However, none of the correlations between game score and frontal theta activity remained significant following Bonferroni correction (all *P*>.003).

**Figure 5:**
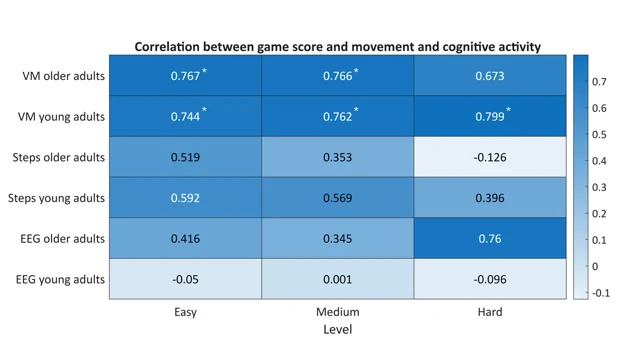
Heatmap of correlations between game score, movement measures, and frontal theta activity during gameplay at different game levels for older and younger participants. Significant correlations following Bonferroni correction (*P*>.003) are marked with an asterisk. EEG: electroencephalogram; VM: vector magnitude.

## Discussion

### Game Scores as Indicators of Movement and Cognitive-Control Demands

This laboratory-based experimental study set out to examine how game scores relate to the amount of movement performed and frontal theta activity during exergaming across different difficulty levels and age groups. The findings showed that the overall amount of movement was strongly and consistently associated with game scores across difficulty levels and age groups, whereas step count demonstrated weaker and less consistent relationships. In contrast, frontal theta activity showed neither statistically significant effects of difficulty level or age group nor a significant interaction between these factors, and exhibited only weak associations with game scores. Taken together, these results suggest that, in this exergame, game performance appears to primarily reflect the amount of movement performed in this specific task rather than cognitive-control demands as indexed by frontal theta activity. Consequently, game scores alone appear to have limited sensitivity to cognitive-control demands in the present exergame and under the specific task conditions investigated, especially if cognitive demands are not strongly or systematically manipulated.

### Game Scores as Proxies for Movement and Cognitive-Control Demands

Game scores are widely used as indicators of performance in exergaming and often function as the primary feedback mechanism for players and clinicians alike. Importantly, game scores reflect how well a player performs within a given set of task constraints, whereas game difficulty level represents a manipulation of those constraints intended to alter physical and cognitive demands [44]. Yet, their usefulness as proxies for physical activity and cognitive-control demands remains underexplored. In our study, game scores were strongly and consistently associated with the overall amount of movement across difficulty levels and age groups, whereas associations with step count were weaker and less consistent. In contrast, frontal theta activity showed no main effects of difficulty level or age group and exhibited only weak associations with game scores, particularly in younger adults. Collectively, these results indicate that, in this exergame, game scores primarily reflect quantitative aspects of movement (ie, how much participants move) rather than cognitive control–related neural engagement. This aligns with previous research showing that different exergame designs and training protocols can lead to distinct physical and cognitive outcomes [41].

This finding should be interpreted in light of previously reported participant experiences. Prior studies have shown that exergaming is perceived as both physically and cognitively demanding, requiring sustained attention and cognitive effort [39,40]. Motivation during gameplay is often driven by tracking progress and receiving rewards for performance, which reinforces engagement and supports skill development [43]. Game design that provides clear and meaningful feedback on progression and effort has been shown to strengthen the sense of mastery and enhance engagement [36,44]. However, the present results suggest a potential mismatch between subjective experiences of cognitive effort and what is actually captured by the scoring system. In the present exergame, game scores were more strongly associated with movement measures than with frontal theta activity, suggesting that they may not fully reflect all task demands experienced by players under the conditions investigated in this study.

The age-related differences observed in the correlation patterns further emphasize this concern. While frontal theta activity showed near-0 associations with game score in younger adults, older adults demonstrated small-to-moderate positive associations across difficulty levels. One possible interpretation is that older adults may rely more on cognitive-control processes to achieve higher scores, potentially as a compensatory strategy for age-related changes in physical or sensorimotor capacity. However, this interpretation should be treated with caution, as the present results do not provide direct support for this mechanism. In particular, the absence of clear differences in frontal theta activity across difficulty levels suggests that increased cognitive-control engagement in older adults was not consistently reflected in the EEG measure. An alternative explanation is that the observed associations may reflect differences in task strategy or efficiency rather than increased cognitive load per se. While previous literature suggests that older adults may show increased cognitive involvement during motor tasks [39], the current findings do not allow us to confirm such compensatory mechanisms within this specific exergame context.

From a rehabilitation and game design perspective, these findings underscore the need for caution when interpreting game scores as indicators of player performance and engagement. If feedback mechanisms such as scores do not scale with cognitive demand or task complexity, they may inadvertently favor low-effort strategies or obscure meaningful gains in cognitive engagement [35,45]. Consequently, reliance on game scores alone may limit the ability to tailor interventions effectively, particularly when cognitive training is an explicit therapeutic goal.

Rather than abandoning scoring systems altogether, the present findings point toward the potential value of integrating game scores into a broader, multidimensional feedback framework. Combining performance scores with objective measures of movement and cognitive activity could provide a more comprehensive representation of player engagement and support more individualized adjustment of difficulty and feedback [46]. Such an approach may help ensure that game scores reflect meaningful progress in both physical and cognitive domains, rather than superficial performance alone, thereby better supporting rehabilitation outcomes and sustained engagement.

### Physical Activity and Game Performance

The results of this study indicate a consistent association between the overall amount of movement (VM) and game score across difficulty levels, alongside a reduction in movement as game difficulty increased. This pattern suggests that, in this exergame, higher scores were largely achieved through greater movement output, reinforcing the notion that game performance is strongly driven by the amount of movement performed rather than cognitive demands. The observed decrease in movement at higher difficulty levels indicates that increasing task complexity, through obstacles or added game constraints, did not elicit a greater amount of movement but instead led to more restricted movement behavior. This finding is consistent with previous work showing that exergame design features, such as increased game speed or the addition of cognitively demanding elements, can alter movement strategies and, in some cases, reduce overall movement output [35,40].

From a rehabilitation perspective, this reduction in movement at higher difficulty levels warrants particular attention. One of the core advantages of exergames lies in their ability to promote high movement dosage through repeated, task‑oriented practice, which is essential for addressing musculoskeletal impairments and supporting functional recovery [20,21]. While increasing difficulty is often intended to enhance challenge and engagement, the present findings suggest that, if not carefully designed, higher difficulty levels may inadvertently constrain movement and undermine this key therapeutic benefit. Moreover, although game scores closely tracked movement quantity, especially in younger adults, step count alone showed weaker associations with performance. This highlights that step count may not adequately capture the complexity and quality of movement in exergames, which frequently involve multidirectional, reactive, and whole‑body actions rather than simple repetitive stepping.

A previous study demonstrated that tailoring game speed to individual player ability can enhance both physical activity and perceived exertion during exergaming [46]. This suggests that the movement patterns observed in this study may have been influenced by fixed difficulty levels that did not account for age-related differences in physical capacity. In the present data, older adults showed more steps at the medium level but fewer steps at the hard level compared with younger adults, despite similar reductions in movement magnitude across difficulty levels. Rather than reflecting differences in step length, this pattern likely indicates a shift toward more cautious or conservative movement strategies when task complexity increases. This is consistent with earlier findings showing that older adults adapt stepping behavior and reduce movement amplitude when exergame demands become more challenging [36,45]. Moreover, the overall decrease in movement at higher difficulty levels suggests that the added obstacles constrained rather than increased physical engagement, and that these elements may not provide the intended cognitive challenge, as frontal theta did not increase with difficulty. Together, these findings highlight the potential value of age‑adaptive and ability‑adaptive game design, which could better balance challenge and movement demands across user groups. Such an approach aligns with motor learning principles emphasizing task‑specific, variable, and sufficiently intense practice to support effective rehabilitation [63]. Hence, by adapting game parameters to individual capabilities, particularly in older adults, exergames may better support physical activity as well as therapeutic outcomes over time.

### Cognitive Activity and Age-Related Differences

Beyond physical engagement, exergames are increasingly recognized for their cognitive benefits, particularly through dual-task elements that require attention, decision-making, and inhibition [14]. In our study, no statistically significant differences in mean frontal theta activity were detected between age groups or across difficulty levels, and associations with performance were generally weak or inconsistent. While older adults exhibited small-to-moderate correlations between frontal theta and game score at lower difficulty levels, a stronger association was observed at the greatest difficulty level. In younger adults, performance appeared to be largely independent of detectable changes in frontal theta.

Previous work has shown that frontal theta can increase during exergaming compared with performing similar movements without cognitive stimuli, and that such engagement can be sustained over time [64]. However, in this study, frontal theta did not vary with difficulty level, suggesting that the cognitive‑control demands imposed by the task may not have been sufficiently differentiated across levels. This interpretation is supported by recent reviews indicating that neural responses to exergaming, including frontal theta activity, vary considerably depending on task design and measurement approaches, and that evidence linking cognitive demand to neurophysiological measures remains limited and heterogeneous [37]. From this perspective, the weak associations observed between game score and frontal theta may reflect characteristics of the specific game design, limited differentiation of cognitive-control demands across difficulty levels, limitations of the measurement approach, or a combination of these factors.

The absence of frontal theta modulation across difficulty levels may also be understood in relation to our previous work using similar exergaming paradigms [35,48]. In those studies, task difficulty was primarily manipulated through changes in game speed, introducing continuous temporal pressure that likely increased sustained attention and cognitive-control demands. In contrast, the current study manipulated difficulty by adding task elements such as multiple targets and inhibitory components, while maintaining a similar overall task structure. This type of manipulation may increase task complexity without substantially altering the level of cognitive-control demand required during gameplay. Consequently, differences in how difficulty is operationalized, such as temporal pressure vs added task elements, may influence whether frontal theta activity is modulated.

Our findings suggest differences in exergame performance between the younger adults and the relatively active older adults included in this study. In particular, younger adults consistently outperformed older adults across all difficulty levels. This may reflect age-related differences in executive functions such as planning and inhibitory control, capacities known to decline with age due to structural and functional brain changes [65,66]. However, interpretations related to specific executive functions such as planning and inhibitory control should be treated with caution, as the present findings do not provide direct evidence that these processes were differentially engaged across task conditions. At the same time, the present findings should be interpreted as reflecting a comparison between younger adults and a relatively active, high-functioning group of community-dwelling older adults, rather than the broader older adult population. Both younger and older participants reported similar levels of weekly physical activity, which may partly reflect differences in how physical activity is perceived and reported across age groups, as well as the inclusion of a broader range of everyday activities among older adults. In addition, the older participants were recruited from recreational exercise groups, likely resulting in a sample with higher-than-average physical activity levels. As such, the present findings should be interpreted in the context of a relatively fit older population.

Physical activity has been shown to mitigate age-related declines in executive function [67,68], and exergaming may offer an added advantage by combining motor and cognitive stimulation in dynamic, unpredictable environments that require real-time decision-making and flexibility [30,69]. While younger adults may not show substantial cognitive benefits due to higher baseline abilities [70], older adults may benefit more from this combined approach. However, in the present study, increasing task difficulty was not associated with statistically significant increases in frontal theta activity. This is in line with previous findings suggesting that older adults do not necessarily exhibit increased frontal theta with added task difficulty [71]. As the different game levels were designed to vary cognitive-control demands through the addition of moving targets and inhibitory elements, the absence of frontal theta differences suggests either that these manipulations did not produce sufficiently differentiated levels of cognitive-control engagement or that frontal theta was not sensitive enough to detect such differences under the present conditions. The absence of clear frontal theta differences is therefore likely explained by a combination of factors. First, the cognitive demands of the exergame may not have been sufficiently distinct across difficulty levels, despite the inclusion of additional elements such as moving targets and inhibitory components. Second, frontal theta was analyzed as an average across entire gameplay trials. Cognitive demands during exergaming are likely intermittent, occurring during brief decision-making events and interspersed with periods dominated by motor execution, and averaging across the full task may therefore reduce sensitivity to transient increases in cognitive engagement. Together, these findings suggest that the observed pattern may reflect characteristics of the task design, the measurement approach, the limited sample size, and the relatively high functional level of the sample, rather than a true absence of cognitive involvement during gameplay.

### Limitations and Future Directions

This is one of very few studies to simultaneously assess movements and frontal theta EEG during exergaming in younger and older adults. However, this study has several limitations that should be acknowledged. First, the sample size was relatively small, which may limit the generalizability of the findings and the statistical power to detect small-to-moderate effects. This is particularly relevant for the EEG analyses, which were based on the subset of participants contributing to the frontal IC cluster. Consequently, nonsignificant findings should not be interpreted as evidence for the absence of age-related or task-related effects, but rather as findings that require confirmation in larger, adequately powered samples. In addition, a convenience sampling approach was used, and the older adults represented an active and high-functioning group. Future studies should therefore include larger and more diverse samples. Second, only a single exergame was examined, and different game designs may elicit different patterns of movements and frontal theta EEG. Future research should explore a broader range of game types and consider longitudinal designs to evaluate changes over time. Third, although extensive preprocessing procedures were applied to reduce movement-related contamination, EEG acquisition during dynamic exergaming remains methodologically challenging. Residual movement-related influences cannot be fully excluded and should be considered when interpreting neural measures obtained during whole-body movement tasks. Fourth, frontal theta was used as a proxy for cognitive-control demands. However, cognition comprises multiple processes that may not be fully captured by this measure alone. Although simplified EEG setups focused on frontal regions may increase feasibility in applied contexts, the present findings suggest that such approaches may require tasks with clearly differentiated cognitive-control demands to elicit meaningful variation. Fifth, frontal theta activity was analyzed as an average across entire gameplay trials. As cognitive demands during exergaming are likely intermittent and embedded within motor activity, this approach may reduce sensitivity to detect transient changes in cognitive engagement. Future studies may benefit from more temporally resolved analyses, including event-related approaches or the investigation of transient theta bursts, which may be better suited to detecting brief episodes of cognitive-control engagement during gameplay. Sixth, movement quality, such as coordination, accuracy, or balance control, was not assessed. Therefore, the extent to which game scores reflect qualitative aspects of movement remains unclear and should be addressed in future work. Finally, although repeated measures general linear models were appropriate for the balanced design used in this study, linear mixed-effects models provide a more flexible framework for modeling individual variability and repeated observations. Future studies with larger samples may benefit from applying such approaches. Overall, the interpretation of correlation strength should be approached with caution, as no universal standards exist, and the observed associations varied across measures and conditions [72].

### Conclusions

This study provides novel insights into how game performance relates to movement and frontal theta EEG during step‑based exergaming across different age groups and difficulty levels. The amount of movement performed was strongly and consistently associated with game scores across difficulty levels and age groups, indicating that performance in this exergame primarily reflects the amount of movement performed in this task, while showing limited association with frontal theta activity under the present game conditions. In contrast, no statistically significant effects of difficulty level or age group were detected for frontal theta EEG, and associations with performance were generally weak or context-dependent. Together, these findings suggest that game scores in this task have limited sensitivity to cognitive-control processes under the present game conditions.

These results highlight the importance of carefully interpreting game performance as an indicator of engagement in exergaming. Reliance on game scores alone may provide an incomplete picture, particularly when cognitive training is an explicit goal. Instead, the findings support the use of multidimensional assessment approaches that integrate movement-based metrics with complementary indicators of cognitive involvement. By combining accelerometry and mobile EEG during whole-body exergaming, this study contributes to a growing but still limited body of research examining the interaction between movement and neurophysiological activity in dynamic, ecologically relevant tasks. Importantly, the results underscore that cognitive engagement should not be assumed based on task complexity alone but depends on how cognitive demands are operationalized within game design.

From a practical perspective, these findings suggest that exergame design should incorporate clearly differentiated and sufficiently challenging cognitive elements if the goal is to engage cognitive-control processes in addition to physical activity. Future research should extend these findings using larger and more diverse samples, as well as task designs that more effectively manipulate cognitive demand. Overall, this study provides a foundation for better understanding what exergame performance reflects and highlights the need for more nuanced approaches to measuring and designing engagement in digital rehabilitation.

## Data Availability

The datasets generated or analyzed during this study are not publicly available due to the inclusion of sensitive and confidential information but are available from the corresponding author upon reasonable request.

## Abbreviations

AMICA: Adaptive Mixture Independent Component Analysis
ASR: automated subspace reconstruction
EEG: electroencephalography
EKG: electrocardiogram
IC: independent component
NTNU: Norwegian University of Science and Technology
VM: vector magnitude
